# Triangulating brain alterations in anorexia nervosa: a multimodal investigation of magnetic resonance spectroscopy, morphometry and blood-based biomarkers

**DOI:** 10.1038/s41398-023-02580-6

**Published:** 2023-08-12

**Authors:** Arne Doose, Friederike I. Tam, Inger Hellerhoff, Joseph A. King, Ilka Boehm, Kim Gottloeber, Hannes Wahl, Annett Werner, Felix Raschke, Brenda Bartnik-Olson, Alexander P. Lin, Katja Akgün, Veit Roessner, Jennifer Linn, Stefan Ehrlich

**Affiliations:** 1 Translational Developmental Neuroscience Section, Division of Psychological and Social Medicine and Developmental Neuroscience, Faculty of Medicine, Technische Universität Dresden, Dresden, Germany; 2 Eating Disorder Research and Treatment Center, Department of Child and Adolescent Psychiatry, Faculty of Medicine, Technische Universität Dresden, Dresden, Germany; 3 Department of Neuroradiology, Faculty of Medicine, Technische Universität Dresden, Dresden, Germany; 4 OncoRay - National Center for Radiation Research in Oncology, Faculty of Medicine and University Hospital Carl Gustav Carus, Technische Universität Dresden, Helmholtz-Zentrum Dresden - Rossendorf, Dresden, Germany; 5 Department of Radiology, Loma Linda University Medical Center, Loma Linda, CA USA; 6 Center for Clinical Spectroscopy, Department of Radiology, Brigham and Women’s Hospital and Harvard Medical School, Boston, MA USA; 7 Center of Clinical Neuroscience, Neurological Clinic, University Hospital Carl Gustav Carus, Faculty of Medicine, Technische Universität Dresden, Dresden, Germany; 8 Department of Child and Adolescent Psychiatry, Faculty of Medicine, University Hospital Carl Gustav Carus, Technische Universität Dresden, Dresden, Germany

**Keywords:** Psychiatric disorders, Neuroscience

## Abstract

The acute state of anorexia nervosa (AN) is associated with widespread reductions in cortical gray matter (GM) thickness and white matter (WM) volume, suspected changes in myelin content and elevated levels of the neuronal damage marker neurofilament light (NF-L), but the underlying mechanisms remain largely unclear. To gain a deeper understanding of brain changes in AN, we applied a multimodal approach combining advanced neuroimaging methods with analysis of blood-derived biomarkers. In addition to standard measures of cortical GM thickness and WM volume, we analyzed tissue-specific profiles of brain metabolites using multivoxel proton magnetic resonance spectroscopy, T1 relaxation time as a proxy of myelin content leveraging advanced quantitative MRI methods and serum NF-L concentrations in a sample of 30 female, predominately adolescent patients with AN and 30 age-matched female healthy control participants. In patients with AN, we found a reduction in GM cortical thickness and GM total N-acetyl aspartate. The latter predicted higher NF-L levels, which were elevated in AN. Furthermore, GM total choline was elevated. In WM, there were no group differences in either imaging markers, choline levels or N-acetyl aspartate levels. The current study provides evidence for neuronal damage processes as well as for increased membrane lipid catabolism and turnover in GM in acute AN but no evidence for WM pathology. Our results illustrate the potential of multimodal research including tissue-specific proton magnetic resonance spectroscopy analyses to shed light on brain changes in psychiatric and neurological conditions, which may ultimately lead to better treatments.

## Introduction

Anorexia nervosa (AN) is a life-threatening eating disorder, which typically begins in adolescence or early adulthood and is characterized by fear of weight gain, an intense pursuit of weight loss through self-starvation and body image distortion. Patients in the acutely underweight state of the disorder show substantial brain changes in multiple measures of brain structure, for which the underlying mechanisms are still largely unclear [1]. Widespread reductions in cortical thickness (CT) with effect sizes larger than in other psychiatric disorders such as schizophrenia and to a lesser degree in white matter (WM) volume have been shown repeatedly [2]. Of note, these CT and WM changes do not persist into (long-term) recovery [3]. Additional closely related brain abnormalities in the acute state of AN include changes in intracortical and WM myelin content as estimated via T1 mapping [4], an advanced quantitative magnetic resonance imaging (qMRI) technique [5], and elevated blood concentrations of neurofilament light (NF-L), a biomarker of neural damage processes [6, 7]. While changes in WM myelin content may reflect oligodendrocyte dysfunction [4] and elevated NF-L may affect cellular size and morphology [6], the underlying mechanisms of the drastic brain changes remain largely unclear.

However, new multimodal approaches, such as the combination of qMRI and blood-derived biomarkers with proton magnetic resonance spectroscopy (^1^H MRS), a non-invasive in-vivo measurement of brain metabolites [8], may help to shed light on the issue [1]. In the current study, we apply these methods together for the first time in AN in attempt to shed new light on the following potential patterns and mechanisms underlying macro- and microstructural brain changes in the disorder.

Neuronal damage processes: Extensive brain cell loss in acute AN is unlikely in light of the relatively fast normalization of brain structure during weight restoration [1, 3]. Recent reports of elevated NF-L concentrations instead point to neuronal damage processes [6, 7]. N-acetyl aspartate is a free amino acid found in the central and peripheral nervous system and commonly measured in ^1^H MRS with its derivative N-acetyl-aspartyl-glutamate as a pooled signal (total N-acetyl aspartate, tNAA) [9]. Reduced levels of tNAA seem to also indicate neuronal damage processes rather than neuronal loss [9].

Oligodendrocyte dysfunction: Dysfunction of cortical oligodendrocytes, which form myelin sheaths around neuronal axons in the central nervous system, may be closely linked to structural brain changes in AN. This is supported by recent “virtual histology” findings, which show that brain regions with high expression of genes specific to oligodendrocytes are especially affected by a reduction of CT [3]. Oligodendrocytes are among the cells most vulnerable to pathological conditions such as oxidative stress, mitochondrial injury and inflammation [10], which may all play a role in AN [11]. Reduced tNAA may also contribute to oligodendrocyte dysfunction and abnormal myelination, since it is a critical source of acetate for myelin lipid synthesis in oligodendrocytes [12]. Furthermore, due to their dependency on lipids for the formation of myelin sheaths [13], oligodendrocytes may exhibit increased cell membrane catabolism and concomitant increased membrane turnover due to decreased fat intake [14] and alterations in lipid metabolism [15] in AN. Using ^1^H MRS, this would also be reflected by elevated concentrations of the total choline (tCho) metabolite pool, which contains contributions of glycerophosphorylcholine, phosphorylcholine and free choline and constitutes a marker for membrane turnover due to the involvement of choline-containing compounds in phospholipid synthesis and degradation [9]. To assess oligodendrocyte dysfunction, qMRI enables quantification of tissue parameters (T1 relaxation) and has been used to link MR signals to differences on a microstructural level [16]. The T1 relaxation time can serve as a proxy for myelin content [17], allowing us to test the hypothesis of oligodendrocyte dysfunction in AN.

We used a multimodal MRI approach including multivoxel ^1^H MRS chemical shift imaging (CSI) to precisely measure tissue-specific metabolite concentrations, CT measures derived from high-resolution T1 weighted (T1w) imaging and quantitative T1 (qT1) mapping in combination with serum NF-L to investigate the underlying mechanisms contributing to brain changes in AN. While almost all previous ^1^H MRS studies in AN chose a single voxel approach suitable for illnesses with regional pathology [18], evidence has been accumulating in favor of global rather than regional structural brain changes in AN [1]. To date, only two studies in AN have used ^1^H MRS CSI [19, 20], which allows for the simultaneous acquisition of many voxels and provides better spatial coverage but raises questions in regard to partial volume effects [21]. To address this issue, the present ^1^H MRS CSI study uses partial volume maps of segmented T1-weighted images in a linear regression model to calculate tissue-specific metabolite concentrations for gray matter (GM) and WM with high precision and statistical power [21]. Assessing tissue-specific metabolite concentrations is essential when investigating their relationship with structural brain changes in AN, which differ in magnitude and trajectory of normalization and may have different underlying mechanisms.

To summarize, our multimodal approach included (1) multivoxel CSI ^1^H MRS with a focus on the tissue-specific concentrations of tNAA and tCho, (2) conventional structural MRI to measure CT and WM volume, (3) qT1 mapping to measure T1 relaxation time as a proxy of myelin content in conjunction with (4) the neuronal damage process marker serum NF-L. We hypothesized that GM and WM tNAA concentrations would be reduced in AN and might be linked to neuronal damage processes and oligodendrocyte dysfunction and possibly associated with CT and WM volume reduction. We further expected GM and WM tCho concentrations to be elevated in AN, signaling increased cell membrane catabolism and turnover, with a link between altered tCho concentrations and oligodendrocyte dysfunction.

## Methods

### Participants

Data of 33 female patients with acute AN and 37 female healthy control participants (HC) were collected, oversampling HC. ^1^H MRS data of three patients with AN and two HC participants did not meet our quality criteria (described below) and were excluded. To account for developmental effects, we used an implementation of the Munkres algorithm for pairwise age-matching [22]. Thus, the final sample consisted of 30 AN (aged 13–21 years) and 30 HC (aged 13–20 years). Patients with AN were admitted to intensive treatment of specialized eating disorder programs at the child and adolescent psychiatry and psychosomatic medicine departments of the University Hospital Dresden and assessed within 96 h of admission to treatment. All protocols received ethical approval by the local Institutional Review Board of the Technische Universität Dresden, and all participants (and, if underage, their guardians) gave written informed consent.

Diagnosis of AN was established using the expert form of the Structured Interview for Anorexia and Bulimia Nervosa (SIAB-EX) [23] and required a body mass index (BMI) below the 10th age percentile (if <15.5 years old) or below 17.5 kg/m² (if >15.5 years old). HC had to be of normal weight, eumenorrhoeic and without any history of psychiatric illness and were recruited through advertisement among middle/high school and university students. The lifetime absence of psychiatric illness in HC was ascertained with the Mini-International Neuropsychiatric Interview for Children and Adolescents (MINI Kid) [24].

Information regarding exclusion criteria was obtained from all participants using the SIAB-EX [23], supplemented by our own semi-structured interview and medical records (Supplement 1). Comorbid diagnoses were taken from medical records and confirmed by an expert clinician.

### Clinical measures

In addition to the SIAB-EX [23], we assessed eating disorder-related psychopathology with the Eating Disorder Inventory-2 (EDI-2) [25] and depressive symptoms with the Beck Depression Inventory-II (BDI-II) [26]. BMI standard deviation scores (BMI-SDS) were computed to provide an age-adjusted index [27]. Study data were managed using Research Electronic Data Capture (REDCap).

### Blood sampling and analyses

Venous blood samples for measurement of plasma leptin, a marker of undernutrition [28], and serum NF-L assessment were collected into vacutainer tubes between 7 and 9 a.m. after an overnight fast. For leptin assessment, aprotinin was added to the plasma samples during blood sampling to prevent protein degradation by serine proteases. Plasma samples were centrifuged immediately and serum samples after 30 min of coagulation time at 6–8 °C (2500×*g* for 15 min, 5 °C), aliquoted, and stored at −80 °C.

Leptin was measured using a commercially available enzyme-linked immunoabsorbent assay (BioVendor Research and Diagnostic Products, Brno, Czech Republic). NF-L was determined using the digital Simoa^TM^ HD-1 Analyzer (Quanterix, Lexington, MA, USA). The study samples for leptin and NF-L assessment were lower than the sample sizes of the group comparisons of ^1^H MRS metabolites (Table 1). Leptin and NF-L concentrations were log-transformed to meet required model form for statistical analysis.

**Table 1:** Study sample: clinical characteristics and structural brain metrics.

	Group, mean (SD)		Analyses		
	AN	HC	*t*	*df*	*p/p*_*adjusted*_
Age (years)	16.1 (2.2)	16.2 (1.8)	−0.31	58.00	0.756/0.756
BMI (kg/m²)	14.1 (1.4)	20.8 (2.1)	−14.19	51.13	<0.001/ < 0.001***
BMI-SDS	−3.61 (1.00)	−0.02 (0.70)	−16.14	58.00	<0.001/ < 0.001***
Minimal lifetime BMI (kg/m²)	14.3 (1.3)	20.0 (1.9)	−11.35	32.39	<0.001/ < 0.001***
EDI-2 (total score)	220.4 (37.6)	135.3 (20.6)	−10.6	41.55	<0.001/ < 0.001***
BDI-II (total score)	29.4 (14.0)	5.1 (5.1)	−8.8	35.22	<0.001/ < 0.001***
Plasma leptin (μg/l)	0.7 (1.3)	11.6 (9.9)	−10.19	29.12	<0.001/ < 0.001***
Cortical thickness (whole brain, mm)	2.39 (0.10)	2.59 (0.08)	−8.05	54.29	<0.001/ < 0.001***
Cerebral white matter volume (cm³)	417806 (44713)	425248 (49350)	−0.60	55.46	0.275/0.330
Serum neurofilament-light (pg/ml)	13.5 (11.7)	4.7 (1.6)	4.50	28.41	<0.001/ < 0.001***
Relaxation time qT1 in gray matter (ms)	1422.7 (20.2)	1416.7 (19.7)	1.13	53.96	0.263/0.330
Relaxation time qT1 in white matter (ms)	832.7 (25.0)	839.3 (22.6)	−1.04	53.45	0.303/0.331

Mean (standard deviation) for each variable are shown for each group. Group differences between AN and HC were tested using the independent samples *t* test. Serum neurofilament-light and plasma leptin concentrations were log-transformed prior to analysis due to deviations from normality. However, for better interpretability, mean (standard deviation) of the raw marker values are displayed in the table. Left-censored leptin concentrations below the lower limit of detection of the applied leptin assay (LOD = 0.20 ng/mL, 17 AN participants in our sample had leptin values below LOD) were imputed using a quantile regression multiple imputation approach for left-censored missing data (QRILC) [58]. P values were adjusted for multiple comparisons (12 variables) using the False Discovery Rate correction method of Benjamini and Hochberg. In the AN group, mean duration of illness was 11.4 (9.0) months. Of the patients at AN, 29/30 (96.7%) were of the restrictive subtype and 1/30 (3.3%) were of the binge/purge subtype. Of the AN participants, 6/30 had one or more psychiatric comorbidities (5/30 current or recent depressive disorder, 1/30 posttraumatic stress disorder, 1/30 specific phobia). Regarding educational attainment and current occupation, 28 of 30 patients with AN were currently enrolled in high school, 1 patient with AN was a highschool graduate without college education, 1 of 30 patients with AN had some college education. In the HC group, 29 of 30 were currently enrolled in high school, 1 of 30 had some college education. In both groups, all participants identified as White. Asterisks denote a significant group difference after multiple comparison correction: *p < 0.05, **p < 0.01, ***p < 0.001.

*AN* patients with anorexia nervosa, *BDI-II* Beck Depression Inventory, *BMI* body mass index, *BMI-SDS* body mass index standard deviation score, *EDI-2* Eating Disorder Inventory-2, *HC* healthy control participants, *SD* standard deviation.

### Magnetic resonance imaging acquisition

**Fig. 1:**
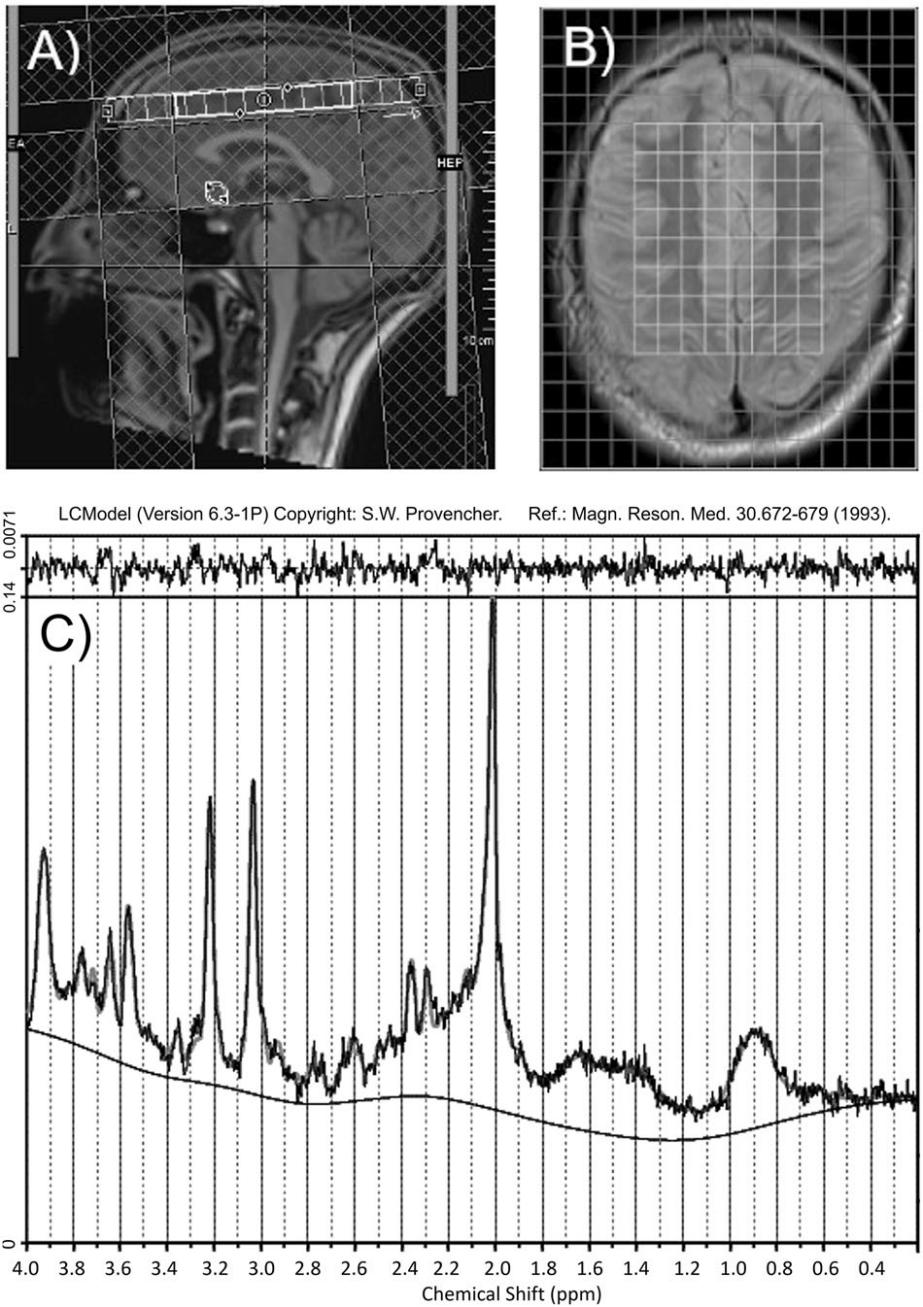
Localization of measured field of view and volume of interest. **A** A two-dimensional 12 mm thick slice (in gray: field of view 130 mm (left-right) × 160 mm (anterior-posterior) × 12.0 mm (inferior-superior), matrix 16 × 16; in white: shim volume) was positioned above the corpus callosum. This is an exemplary magnet resonance imaging scan of the head of one of the authors who gave consent for publication. **B** Volume of interest: middle 8 × 8 voxels, 90 mm (anterior-posterior) × 75 mm (left-right) × 12.0 mm (inferior-superior), voxel size 10.0 mm × 8.1 mm × 12.0 mm. **C** LCModel fitted spectrum of one exemplary voxel of one exemplary participant used in our statistical analysis.

All imaging measurements were conducted in one session on a 3 T (123.25 MHz) Magnetom Prisma Scanner (Siemens Healthineers, Erlangen, Germany) with a 32-channel head coil between 2 p.m. and 4 p.m. After a localizer sequence, high-resolution three-dimensional T1-weighted structural scans were acquired with a MP2RAGE sequence [5] with 1 mm isotropic resolution (repetition time (TR) 5000 ms, echo time (TE) 2.9 ms, TI1/TI2 700 ms/2500 ms, FA = 4/9). 2D Multivoxel ^1^H MRS CSI was acquired with the SIEMENS CSI spin echo sequence (TR 1700 ms, TE 30 ms, no acceleration factor, Prescan Normalize, Hamming filter (50%)) with 4 averages (see “Minimum Reporting Standards for in vivo Magnetic Resonance Spectroscopy (MRSinMRS) checklist” from the consensus paper [29] in Supplementary Information (Supplement 1)). The acquisition was repeated using a single average and without water suppression for eddy current correction and water-scaling. Figure 1 depicts the volume of interest (VOI) and field of view, which included the dorsal anterior cingulate cortex (dACC) and was angled so that it did not cover the subarachnoid space while including a maximum volume of GM/WM brain tissue for later regression analyses. For positioning, we used a vendor-provided automatic voxel positioning (SIEMENS auto-align) technique as described by Dou et al. [30] followed by manual correction in the event of significant anatomical deviations. Outer volume suppression bands were placed around the VOI. For B_0_ shimming, we used the scanners GRE 3D B_0_ shim routine and then manually adjusted to achieve a mean full width half maximum (FWHM) of 17.1 Hz (standard deviation = 1.7 Hz).

### Data processing

GM and WM ^1^H MRS metabolite concentrations for each participant were obtained using the VDI libraries [31] in Matlab R2018a (version 9.4). Briefly, MP2RAGE INV2 images (set of images with longer inversion time of 2500 ms) were segmented with SPM12 (https://www.fil.ion.ucl.ac.uk/spm/ [32, 33]) running on Matlab R2018a (version 9.4). Segmentation resulted in tissue probability maps for GM, WM, and cerebrospinal fluid. Resulting cerebrospinal fluid, WM and GM maps were registered to the ^1^H MRS VOI using VDI libraries. Within the VDI libraries, the tissue volume fraction for each voxel was calculated in the VOI separately for each participant, and participant-specific global GM and WM metabolite ratios to tCr (signal contributions of creatine and phosphocreatine) were calculated using linear regression [21]. LCModel (Version 6.3, http://s-provencher.com/lcmodel.shtml) was used for ^1^H MRS metabolite quantification using the default, simulated LCModel basis set and water-scaling. The following metabolites were included in the analysis: tNAA (signal contributions of N-acetyl aspartate and N-acetyl-aspartyl-glutamate), tCho (signal contributions of glycerophosphorylcholine, phosphorylcholine and free choline), mIns (myo-inositol) and Glx (signal contributions of glutamine and glutamate). Metabolite concentrations were reported in ratios to tCr.

All outer voxels were excluded to avoid large chemical shift displacement artifacts. For the remaining voxels (matrix 6 x 6), we performed a semi-automated quality control approach. In accordance with the recommendations of Wilson et al. [34], voxels with a FWHM higher than 0.1 ppm and/or a signal-to-noise ratio of less than 3 were flagged with an in-house software and excluded. The spectra of all the remaining voxels were visually inspected by a single trained rater (to avoid bias due to inter-rater variability) using a standardized workflow. Visual spectra inspection focused on the shape of major landmark peaks (tCho, tCr, tNAA), the identification of baseline distortions and effects of spurious echoes as well as other artifacts such as lipid contamination. Furthermore, the Cramér‐Rao lower bounds of the major metabolites as well as the LCModel Diagnostics output were checked. All questionable spectra were discussed with a supervising ^1^H MRS expert before inclusion or exclusion. In total, 113/2340 (4.8%) of all voxels were excluded from the analysis. This included all voxels (*n* = 108) of three measurements due to severe artifacts in a large proportion of voxels per measurement (*n* = 1 AN, *n* = 2 HC), one voxel each from three participants (*n* = 2 AN, *n* = 1 HC) and two voxels from one HC participant.

To obtain CT, we used the standard automated FreeSurfer (version 7.1.1) workflow (Cambridge, Massachusetts, USA, http://surfer.nmr.mgh.harvard.edu) [35] including motion correction, realignment, normalization, Talairach transformation and registration to the Destrieux Atlas [36]. In addition, we applied a brain extraction step using the following procedures: First, FSL BET (FMRIB, Oxford, UK [37]) was used to generate masks on MP2RAGE INV2 images (TI = 2500 ms), which naturally have low background noise. Binarized masks were multiplied with the UNI images (T1w images, combination of weighted INV1 and INV2 images, but with high background noise [5]). Finally, these skull-stripped UNI images were fed into the FreeSurfer stream to obtain CT [38, 39]. To assure the quality of the surface reconstruction, all reconstructed images were visually inspected by a trained examiner. We extracted CT from a region of interest (ROI) consisting of the middle-anterior part of the bilateral cingulate gyrus and sulcus as defined in the Destrieux Atlas [36], which shows high overlap with our VOI of the main ^1^H MRS analyses (Supplementary Fig. S1), as well as from the whole brain (Table 1). Cerebral WM volume from the whole brain was also calculated with the standard FreeSurfer procedures [35, 40] as previously described [41].

qT1 maps were calculated directly on-site after obtaining the MP2RAGE scan according to SIEMENS standard using syngo MapIt (Siemens Healthineers, Erlangen, Germany). The qT1 maps were registered to the Destrieux Atlas with Freesurfer [42] with mean T1 relaxation times for whole brain GM, and GM within the ROI was calculated using an in-house Python script (Version 3.8).

### Statistical analysis

Statistical analyses were performed using R (Version 4.0.4) and IBM SPSS Statistics for Windows, version 27.0 (IBM Corp., Armonk, NY). Independent *t* tests were applied for group comparisons between AN and HC (^1^H MRS metabolite concentrations in GM and WM, structural brain metrics CT and WM volume, NF-L (log-transformed to meet assumptions for parametric testing), T1 relaxation time (qT1) in cortical GM and WM). To investigate the underlying mechanisms of these brain changes, we defined the following linear regression models to test the hypotheses outlined above in the case that group differences in the respective tissue-specific ^1^H MRS metabolite concentration were found:

1. Hypothesis: Altered GM or WM tNAA/tCr concentrations are linked to group differences in NF-L concentrations or CT/WM volume (including “estimated total intracranial volume” (eTIV)) as a covariate):

$$1{{{\mathrm{a}}}}_{{{{\mathrm{GM}}}}}\!\!:\,Serum\,NF-L = \beta _1 \cdot tNAA/tCr_{GM} + \beta _2 \cdot Group + \beta _3 \cdot {{{\mathrm{Interaction}}}}\left( {tNAA/tCr_{GM}|Group} \right) + \varepsilon$$

$$1{{{\mathrm{a}}}}_{{{{\mathrm{WM}}}}}\!\!:\,Serum\,NF-L = \beta _1 \cdot tNAA/tCr_{WM} + \beta _2 \cdot Group + \beta _3 \cdot {{{\mathrm{Interaction}}}}\left( {tNAA/tCr_{WM}|Group} \right) + \varepsilon$$

$$1{{{\mathrm{b}}}}_{{{{\mathrm{GM}}}}}\!\!:\,CT = \beta _1 \cdot tNAA/tCr_{GM} + \beta _2 \cdot Group + \beta _3 \cdot {{{\mathrm{Interaction}}}}\left( {tNAA/tCr_{{{{{GM}}}}}|Group} \right) + \varepsilon$$

$$1{{{\mathrm{c}}}}_{{{{\mathrm{WM}}}}}\!\!:\,WM\,Vol = \beta _1 \cdot tNAA/tCr_{WM} + \beta _2 \cdot Group + \beta _3 \cdot {{{\mathrm{Interaction}}}}\left( {tNAA/tCr_{WM}|Group} \right) + eTIV + \varepsilon$$

2. Hypothesis: Altered GM or WM tNAA/tCr or tCho/tCr concentrations are linked to oligodendrocyte dysfunction:

$$2{{{\mathrm{a}}}}_{{{{\mathrm{GM}}}}}\!\!:\,qT1_{GM} = \beta _1 \cdot tNAA/tCr_{GM} + \beta _2 \cdot Group + \beta _3 \cdot {{{\mathrm{Interaction}}}}\left( {tNAA/tCr_{GM}|Group} \right) + \varepsilon$$

$$2{{{\mathrm{a}}}}_{{{{\mathrm{WM}}}}}\!\!:\,qT1_{WM} = \beta _1 \cdot tNAA/tCr_{WM} + \beta _2 \cdot Group + \beta _3 \cdot {{{\mathrm{Interaction}}}}\left( {tNAA/tCr_{WM}|Group} \right) + \varepsilon$$

$$2{{{\mathrm{b}}}}_{{{{\mathrm{GM}}}}}\!\!:\,qT1_{GM} = \beta _1 \cdot tCho/tCr_{GM} + \beta _2 \cdot Group + \beta _3 \cdot {{{\mathrm{Interaction}}}}\left( {tCho/tCr_{GM}|Group} \right) + \varepsilon$$

$$2{{{\mathrm{b}}}}_{{{{\mathrm{WM}}}}}\!\!:\,qT1_{WM} = \beta _1 \cdot tCho/tCr_{WM} + \beta _2 \cdot Group + \beta _3 \cdot {{{\mathrm{Interaction}}}}\left( {tCho/tCr_{WM}|Group} \right) + \varepsilon$$

Further, we calculated Pearson correlations to explore associations between ^1^H MRS concentrations and BMI-SDS, age, BDI-II total score, EDI-2 total score, duration of illness (in the AN group), and log-transformed leptin. Statistical significance was defined as *p* < 0.05, with *p* values adjusted for multiple comparisons using the False Discovery Rate correction method [43] where appropriate.

## Results

Demographic and clinical characteristics are summarized in Table 1. There was no age difference between the AN and the HC groups. As expected, patients with AN had lower BMI-SDS and leptin concentrations and higher levels of psychopathology (EDI-2, BDI-II) than HC. None of the participants were on psychoactive medication.

**Fig. 2:**
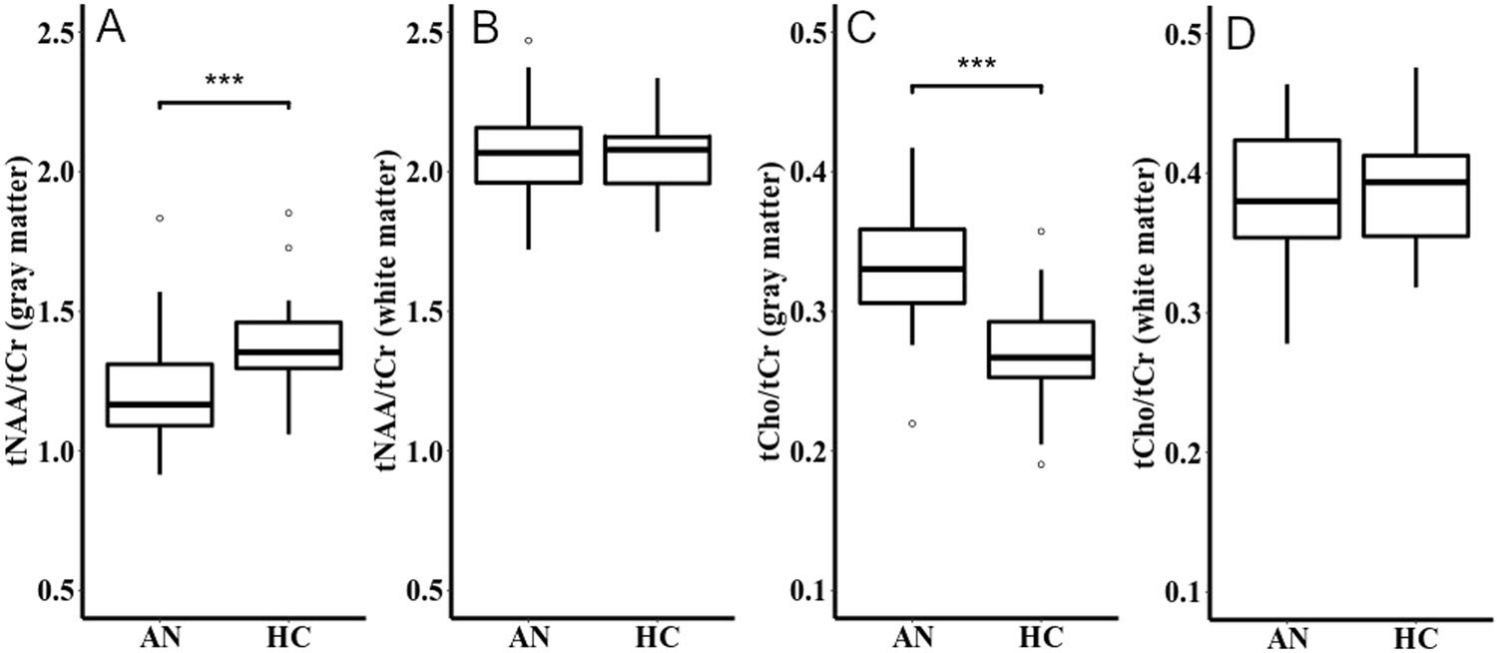
Ratios of total N-acetyl-aspartate and total choline to total creatine in gray and white matter. Box plots showing the median, upper and lower quartile, outliers (depicted as circles, values deviating more than 1.5 times the interquartile range from the upper or lower quartile) and the kernel probability density of the data at different values. Asterisks denote a significant group difference after multiple comparison correction: *p < 0.05, **p < 0.01, ***p < 0.001. **A** Total N-acetyl-aspartate/total creatine ratios in gray matter; AN: *Mean* = 1.21, SD = 0.20, range 0.91 to 1.83; HC: Mean = 1.38, SD = 0.16, range 1.06 to 1.85. Independent *t* tests showed lower total N-acetyl-aspartate/total creatine ratios in gray matter in the AN group than in the HC group, *t*(55.68) = −3.58, *p* < 0.001 (adjusted *p* < 0.001), *Cohen’s d* = 0.94. **B** Total N-acetyl-aspartate/total creatine ratios in white matter; AN: Mean = 2.06, SD = 0.17, range 1.72 to 2.47; HC: Mean = 2.06, SD = 0.13, range 1.78 to 2.34. Independent *t* tests showed no group difference regarding total N-acetyl-aspartate/total creatine ratios in white matter between the AN and the HC group, *t*(54.30) = −0.08, *p* = 0.470 (adjusted *p* = 0.627), *Cohen’s d* = 0.02. **C** Total choline/total creatine ratios in gray matter; AN: Mean = 0.33, SD = 0.04, range 0.22 to 0.42; HC: Mean = 0.27, SD = 0.04, range 0.19 to 0.36. Independent t-tests showed higher total choline/total creatine ratios in gray matter in the AN group than in the HC group, *t*(57.13) = 5.86, *p* < 0.001, (adjusted *p* < 0.001), *Cohen’s d* = −1.54. **D** Total choline/total creatine ratios in white matter; AN: Mean = 0.38, SD = 0.05, range 0.28 to 0.46; HC: Mean = 0.39, SD = 0.04, range 0.32 to 0.48. Independent *t* tests showed no group difference regarding choline in white matter between the AN and the HC group, *t*(55.87) = −0.66, *p* = 0.746 (adjusted *p* = 0.746), *Cohen’s d* = 0.17. This figure was created with R [57]. AN patients with anorexia nervosa, HC healthy control participants, tNAA total N-acetyl-aspartate concentrations (signal contributions of N-acetyl aspartate and N-acetyl-aspartyl-glutamate concentrations), tCho total choline concentration (signal contributions of glycerophosphorylcholine, phosphorylcholine and free choline), tCr total creatine concentration (signal contributions of creatine and phosphocreatine).

Compared to HC, the AN group showed a reduction of GM tNAA/tCr concentrations (*Cohen’s d* = 0.94) and an elevation of GM tCho/tCr concentrations (*Cohen’s d* = −1.54) (Fig. 2). There were no group differences between AN and HC for WM tNAA/tCr and tCho/tCr (Fig. 2).

As expected, we observed a reduction of CT in the AN group relative to HC, both in the whole brain (*Cohen’s d* = 2.15, Table 1), and in the GM ROI (*Cohen’s d* = 1.89, Supplementary Fig. S1), which positively correlated with BMI-SDS. In contrast, no group difference was evident for WM volume (Table 1). As expected, we found elevated serum NF-L concentrations in the AN group relative to HC (*Cohen’s d* = −1.32, Table 1) indicative of neuronal damage processes. However, no group difference was detected for the T1 relaxation time qT1 either in cortical GM (*Cohen’s d* = −0.31, Table 1) or WM (*Cohen’s d* = 0.28, Table 1).

Because group differences in ^1^H MRS metabolite concentrations were only found in GM but not WM, the linear regression models 1a and 1b (but not 1c) were calculated for GM, and the linear regression models 2a and 2b were calculated for qT1 in GM but not WM. The overall regression analysis of model 1a_GM_ was significant (*R²* = 0.30, *F(3,47)* = 6.79, *p* < 0.001). The coefficients tNAA/tCr (*β*_*1*_ = −0.50, *p* = *0.038*) and group (HC/AN) (*β*_*2*_ = −0.67, *p* = *0.031*) both predicted higher NF-L concentrations, but the interaction of group and tNAA/tCr (*β*_*3*_ = 0.52, *p* = 0.095) showed no significance. Model 1b_GM_ was also significant (*R²* = 0.48, *F(3,54)* = 16.50, *p* < 0.001). Group (HC/AN) predicted CT (*β*_*2*_ = 0.23, *p* < 0.001), while tNAA/tCr (*β*_*1*_ = 0.00, *p* = 0.998) and the interaction of group and tNAA/tCr (*β*_*3*_ = 0.03, *p* = 0.525) showed no predictive value. Models 2a_GM_ (*R²* = 0.04, *F(3,52)* = 0.69, *p* = 0.563) and 2b_GM_ (*R²* = 0.01, *F(3,52)* = 0.19, *p* = 0.904) were not significant.

The exploratory analysis of Glx/tCr and mIns/tCr showed an elevation of Glx/tCr in GM in the AN group (GM Glx/tCr *Cohen’s d* = −0.93) and reduced mIns/tCr in GM and WM for AN (GM mIns/tCr *Cohen’s d* = 0.78, WM mIns/tCr *Cohen’s d* = 0.79) (Table 2). For WM Glx/tCr, no group difference emerged (Table 2).

**Table 2:** Ratios of myoinositol/total creatine and glutamine/glutamate/total creatine in gray and white matter.

		Group, mean (SD)		Analyses			
		AN	HC	*t*	*df*	*p/p*_adjusted_	*Cohen’s d*
Gray matter	mIns/tCr	0.85 (0.08)	0.91 (0.07)	−2.97	56.64	*p* = 0.004/0.006**	0.78
	Glx/tCr	2.18 (0.19)	1.98 (0.23)	3.56	56.56	*p* < 0.001/0.003**	−0.93
White matter	mIns/tCr	0.74 (0.08)	0.81 (0.10)	−3.01	54.49	*p* = 0.004/0.006**	0.79
	Glx/tCr	1.30 (0.13)	1.32 (0.14)	−0.66	57.83	*p* = 0.514/0.514	0.17

Metabolite ratios. Mean (standard deviation) for each metabolite are shown for each group. Group differences between AN and HC were tested using the independent samples t test. *p* values were adjusted for multiple comparisons (4 variables) using the False Discovery Rate correction method of Benjamini and Hochberg. Asterisks denote a significant group difference after multiple comparison correction: *p < 0.05, **p < 0.01, ***p < 0.001. AN patients with anorexia nervosa, *Glx* glutamine and glutamate concentrations (pooled signal), *HC* healthy control participants, *mIns* myoinositol concentration, *tCr* total creatine concentration (signal contributions of creatine and phosphocreatine).

The correlation analyses exploring relationships between the altered ^1^H MRS metabolite concentrations (GM tNAA/tCr, GM tCho/tCr, GM Glx/tCr, GM mIns/tCr and WM mIns/tCr) and clinical variables as well as log-transformed leptin revealed no associations in the AN or the HC group (Supplementary Table S1).

Significant results of the group comparisons of all ^1^H MRS metabolite concentrations were confirmed by supplementary analyses excluding AN participants with binge-purge subtype (*N* = 1) and those with psychiatric comorbidity (*N* = 6, Supplementary Table S2).

## Discussion

In the present study, we aimed to shed light on the potential patterns and mechanisms underlying the substantial brain changes commonly seen in acutely ill patients with AN [3] by using a multimodal approach including multivoxel ^1^H MRS to investigate tissue-specific brain metabolite profiles, conventional structural MRI, qT1 mapping, and the neuronal damage process marker serum NF-L. Our finding of reduced GM tNAA/tCr in patients, which predicted higher NF-L in the regression analysis, implies that neuronal damage processes might constitute a dominant process. Also, in line with predictions, we found GM tCho/tCr concentrations to be elevated in AN, suggestive of increased membrane lipid catabolism and turnover. In WM, however, we found no group differences for either tNAA/tCr or tCho/tCr.

As outlined in the introduction, we expected that the applied multimodal approach would help elucidate whether macrostructural brain changes in AN might be at least partially attributable to either neuronal damage processes and/or oligodendrocyte dysfunction. Our findings delivered evidence in support of both possibilities. Regarding the neuronal damage hypothesis, conventional structural MRI analyses showed reduced CT, and serum analysis confirmed elevated NF-L in AN compared to the HC group. Moreover, GM tNAA/tCr was significantly lower in AN participants than in HC, and a negative association with NF-L was evident. Together, this pattern of results is in line with recent ^1^H MRS studies, which have interpreted tNAA/tCr reduction as a sign of reversible neuronal damage processes rather than adopting its earlier interpretation as a marker of irreversible neuronal loss [12]. Since tNAA/tCr is likely synthesized in neuronal mitochondria, a reduced tNAA/tCr concentration may also be a marker of mitochondrial dysfunction or energy state [12]. Our findings might be mediated by such a mitochondrial dysfunction, which has been shown in leukocytes in AN and may be closely related to oxidative stress [11]. The lack of an association between GM tNAA/tCr concentrations with CT in the AN group may be explained by the multitude of possible influencing factors on cortical thinning besides neuronal damage processes, such as macronutrient and micronutrient deficiencies, hormonal changes and hydration status [1], as well as the complex timeline of GM changes. A link between neuronal damage processes and CT reduction is further supported by recent results specifically pointing to larger differences in CT between AN and HC in regions with high expression of genes specific to pyramidal neurons [3].

Regarding the oligodendrocyte dysfunction hypothesis, altered tNAA/tCr concentrations may be indicative of changes in oligodendrocyte function and myelin lipid metabolism, as tNAA/tCr provides acetate for myelin synthesis [12]. Additionally, our finding of elevated GM tCho/tCr in AN could indicate increased membrane lipid catabolism and turnover, which may be of particular importance in myelin − a multilamellar membrane consisting of 40 or more lipid bilayers [13]. However, in our study population, no group difference between AN and HC was found for T1 relaxation time in GM, which has been suggested as a marker for cortical myelin content, and there was no significant association between tNAA/tCr or tCho/tCr and T1 relaxation time in the AN group. While these findings do not imply a strong relationship between tNAA/tCr and tCho/tCr changes and decreased myelination in AN, it should be taken into consideration that T1 relaxation time may be partly influenced by iron and not exclusively by myelin content [44]. It cannot be ruled out that this might mask myelination changes, especially as there is evidence for altered iron metabolism in AN [45].

Our main ^1^H MRS finding of reduced GM tNAA/tCr is in line with the findings of two previous single voxel ^1^H MRS studies in frontal GM and the left insular cortex [18, 46]. In contrast, most single voxel ^1^H MRS studies found no group difference between AN and HC for tNAA/tCr [47–50], assessed at different or not further specified timepoints during treatment. A ^1^H MRS CSI study (VOI positioning similar to present study) reported no group difference in the parietal part of the VOI, but elevated GM tNAA/tCr in the frontal part [19]. The observed increase in GM tCho/tCr is in accordance with the findings of the aforementioned ^1^H MRS CSI study, which found elevated tCho/tCr concentrations in GM but not WM [19]. While ^1^H MRS single voxel studies measuring tCho/tCr or tCho showed mixed results [18, 46, 47, 49, 50], in a ^31^P MRS study, Blasel et al. [19] found a significant elevation of glycerophosphoethanolamine and a trend to elevated glycerophosphocholine – both phospholipid catabolites – in one of their target brain regions. These changes in membrane phospholipid catabolism could be related to low lipid intake in AN [19].

The exploratory analysis of the metabolites mIns/tCr and Glx/tCr revealed elevated Glx/tCr concentrations in AN in GM but not WM and reduced mIns/tCr in GM and WM. The pooled signal Glx/tCr can be quantified with high accuracy, while its constituents glutamate and glutamine only become separable at a magnetic field strength of 7 T or higher [9]. While our result of a Glx/tCr elevation in GM in AN is challenging to interpret, it may play a role in neuronal damage processes. Blasel et al. [19], who reported similar findings, argued that elevated glutamate, which may promote cell-damaging effects on neuronal tissue through excitotoxicity, may induce cell membrane degradation. However, single voxel ^1^H MRS studies at 1.5 T or 3 T found no group difference between AN and HC [18, 47, 49–51] or reduced Glx concentrations in AN [46, 50]. Results from a pilot investigation at 7 T (n = 13 patients with AN) indicated a reduction of glutamate in AN but no group difference between AN and HC for glutamine [48]. Unfortunately, comparison with our results is limited, as this study included patients with AN at different stages of treatment and many were taking psychotropic medication [48], which may act on the glutamergic system [52]. Our result of reduced mIns/tCr in GM and WM in AN is in line with previous studies that reported reduced levels of mIns or ratios of mIns/tCr in AN compared to HC in WM and/or GM [20, 46, 50, 51, 53]. MIns is considered a marker of glial cell integrity as it is mainly located in astrocytes and microglia cells [54] and also has a function in axonal-glial signaling [51]. Impaired astrocyte function has previously been shown in rodent models of AN, and a reduction in astrocytes has been associated with changes in brain volume in rodent models [55, 56]. Therefore, the underlying mechanisms of mIns reduction in AN may include trophic changes in the brain or an imbalance in the second messenger signaling system [20].

When considering our findings, some limitations have to be taken into account. First, some of the metabolites of interest, such as Glx, could only be quantified as pooled signals in this 3 T study. Future studies at higher magnetic field strength may allow for a separate quantification of these resonances, which may facilitate the interpretation of metabolite changes. Second, since our sample consisted of individuals aged 12 to 21 years, developmental effects may be a confounding factor, which we tried to address by using pair-wise age-matching. Third, the ROI from which CT was extracted did not correspond exactly with the ^1^H MRS VOI but showed a high overlap. Finally, as all participants were female and identified as White, our findings cannot be generalized to all individuals with AN.

In conclusion, the current multimodal neuroimaging study sheds new light on the potential patterns and mechanisms underlying substantial brain changes in AN. Our finding of an AN-related reduction of GM tNAA/tCr and its association with serum NF-L implies neuronal damage processes as a prominent mechanism. Furthermore, the observed elevated GM tCho/tCr concentrations are suggestive of increased membrane lipid catabolism and turnover. Together, our results illustrate the potential multimodal studies including tissue-specific ^1^H-MRS analyses have to unravel thus far largely unexplained brain changes in patients with AN. Ultimately, research following the model applied in our study may help in the development of novel neuroprotective treatments in the acute phase of the disorder.

### Supplementary information

supplements
