# Supplementary material for: Triangulating brain alterations in anorexia nervosa: a multimodal investigation of magnetic resonance spectroscopy, morphometry and blood-based biomarkers
Source: Transl Psychiatry. 2023 Aug 12;13:277. doi: 10.1038/s41398-023-02580-6 (PMC10423271; doi:10.1038/s41398-023-02580-6)
Supplement: Supplementary file 1 — supplements [file 41398_2023_2580_MOESM1_ESM.docx]

***Supplemental Information for***

***Triangulating brain alterations in anorexia nervosa: a multimodal investigation of magnetic resonance spectroscopy, morphometry***

***and blood-based biomarkers***

**Supplement 1: Methods**

**Exclusion criteria**

Participants of all groups were excluded if they had a history of any of the following diagnoses: organic brain syndrome, schizophrenia, substance dependence, psychosis not otherwise specified, bipolar disorder, bulimia nervosa, or binge-eating disorder. Further exclusion criteria for all participants were an IQ below 85; current substance abuse; inflammatory, neurologic or metabolic illness; chronic medical or neurological illness that could affect appetite, eating behavior or body weight; clinically relevant anemia; pregnancy or breast feeding. Psychoactive medication within four weeks before the study (except for certain antidepressants) were additional exclusion criteria for all groups.

**Magnetic Resonance Spectroscopy reporting checklist**^1^

| 1. Hardware | |
| --- | --- |
| a. Field strength [T] | 3 T (123.25 Mhz) |
| b. Manufacturer | Siemens |
| c. Model (software version if available) | MAGNETOM Prisma (VE11) |
| d. RF coils: nuclei (transmit/receive), number of channels, type, body part | ^1^H, head, 32 channels |
| e. Additional hardware | None |
| 2. Acquisition | |
| a. Pulse sequence | SIEMENS CSI spin echo |
| b. Volume of Interest (VOI) locations | Above the corpus callosum, including dorsal the anterior cingulate cortex (see Figure 1) |
| c. Nominal VOI size [cm^3^, mm^3^] | 75 x 90 x 12 mm³ |
| d. Repetition Time (T_R_), Echo Time (T_E_) [ms, s] | T_R_ 1700 ms, T_E_ 30 ms |
| e. Total number of excitations or acquisitions per spectrum in time series for kinetic studies  i. Number of Averaged spectra per time-point  ii. Averaging method (e.g. block-wise or moving average)  iii. Total number of spectra (acquired / in time-series) | 4 averages |
| f. Additional sequence parameters (spectral width in Hz, number of spectral points, frequency  offsets)  If STEAM:, Mixing Time (TM)  If MRSI: 2D or 3D, FOV in all directions, matrix size, acceleration factors, sampling method | 2D: 130 x 160 x 12 mm³ FOV, matrix size 16 x 16, no acceleration factor, Prescan Normalize, Hamming (50%) |
| g. Water and fat suppression method | Water sat., 50 Hz; placement of six outer volume suppression bands |
| h. Shimming Method, reference peak, and thresholds for “acceptance of shim” chosen | Automated Siemens GRE 3D B_0_ field mapping technique followed by manual adjustment < 20 Hz, Mean FWHM=17.1 (SD=1.7) |
| i. Triggering or motion correction method (respiratory, peripheral, cardiac triggering, incl. device | None |
| 3. Data analysis methods and outputs | |
| a. Analysis software | LCModel version 6.3 |
| b. Processing steps deviating from quoted reference or product | None |
| c. Output measure (e.g. absolute concentration, institutional units, ratio) | Institutional units and ratios (subanalysis) |
| d. Quantification references and assumptions, fitting model assumptions | LCModel default basis set (simulated) |
| 4. Data Quality | |
| a. Reported variables (SNR, Linewidth (with reference peaks)) | SNR: 25.54 ± 2.4 (20-32), FWHM: 0.033 + 0.004 (0.027-0.046) ppm as reported by LCmodel |
| b. Data exclusion criteria | SNR<3, FWHM>0.1ppm, substantial artifacts detected in visual inspection |
| c. Quality measures of postprocessing Model fitting (e.g. CRLB, goodness of fit, SD of residual) | CRLB (%SD): NAA 2.47 ± 0.21 (2.08-2.89), tCho 3.05 ± 0.20 (2.31-3.83), tCr 2.62 ± 0.20 (2.17-3.11), Ins 5.14 ± 0.52 (4.33-7.44), Glx 7.78 ± 0.64 (6.56-9.22) |
| d. Sample Spectrum | see Figure 1 |


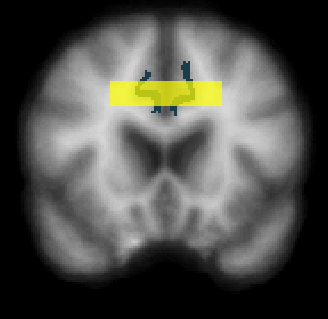

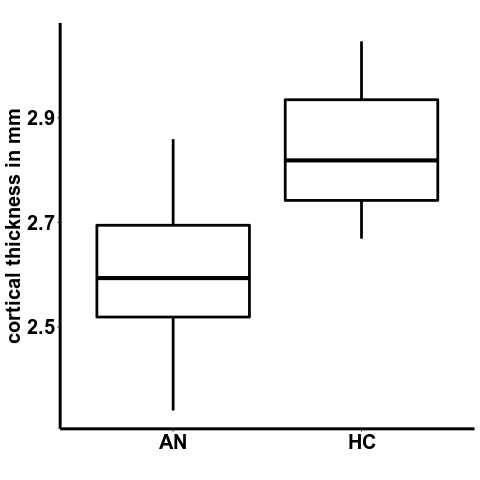


**B**

**A**

**Figure S1: Cortical thickness in the middle-anterior part of the cingulate gyrus and sulcus**

Panel A: Mean cortical thickness reported in mm in the middle-anterior part of the cingulate gyrus and sulcus (defined in the Destrieux Atlas ^2^). AN: *Mean*=2.60, *standard deviation*=0.14, *range* 2.34 to 2.86; HC: *Mean*=2.84, *standard deviation*=0.11, *range* 2.67 to 3.05. Independent t-tests showed lower cortical thickness in the AN group than in the HC group, *t*(52.78)=-7.07, *p<*0.001, *Cohen’s d*=1.89. A linear correlation between cortical thickness and standardized body mass index was shown in the AN group (*r*=0.45, *p*=0.015) but not in the HC group (*r*=-0.16, *p*=0.415). Cortical thickness measures were extract with the standard FreeSurfer procedures as in Bernardoni et al. ^3^. Surface reconstruction for each hemisphere included tessellation of the gray matter-white matter boundary, automated topology correction, and surface deformation following intensity gradients to optimally place the gray-white and gray-cerebrospinal fluid borders at the location where the greatest shift in intensity defines the transition to the other tissue class. The resulting surfaces were then used to calculate cortical thickness for each vertex as the minimal distance from the pial surface to boundary of gray and white matter. Extracted measures were smoothed with a Gaussian kernel with a full width at half a maximum of 10 mm. Panel B: Coronal plane of the FreeSurfer *fsaverage* image including the overlap of the volume of interest used in the main analysis (yellow) and the middle-anterior part of the cingulate gyrus and sulcus in the left and right hemisphere (blue) used in the supplementary correlation analyses of cortical thickness with gray matter total N-acetyl-aspartate and total choline. Abbreviations: AN, patients with anorexia nervosa; HC, healthy control participants.

**Supplement 2: Results**

**Table S1. Correlations of total N-acetyl-aspartate, total choline, glutamine/glutamate and myoinositol concentrations in gray matter with clinical variables**

|  |  | BMI-SDS | Age | BDI-II | EDI-2 | Duration of illness | Plasma leptin* |  |
| --- | --- | --- | --- | --- | --- | --- | --- | --- |
| AN | tNAA/tCr in gray matter | *r*=0.16  *p*=0.406  *p_adjusted_*=0.995 | *r*=0.15  *p*=0.434  *p_adjusted_*=0.995 | *r*=0.19  *p*=0.331  *p_adjusted_*=0.995 | *r*=-0.01  *p*=0.980  *p_adjusted_*=0.995 | *r*=-0.07  *p*=0.757  *p_adjusted_*=0.995 | *r*=0.32  *p*=0.129  *p_adjusted_*=0.831 |  |
|  | tCho/tCR in gray matter | *r*=-0.01  *p*=0.969  *p_adjusted_*=0.995 | *r*=0.14  *p*=0.485  *p_adjusted_*=0.995 | *r*=-0.17  *p*=0.399  *p_adjusted_*=0.995 | *r*=-0.13  *p*=0.416  *p_adjusted_*=0.995 | *r*=-0.01  *p*=0.976  *p_adjusted_*=0.995 | *r*=-0.04  *p*=0.844  *p_adjusted_*=0.995 |  |
|  | Glx/tCR in gray matter | *r*=-0.32  *p*=0.086  *p_adjusted_*=0.860 | *r*=-0.50  *p*=0.006  *p_adjusted_*=0.180 | *r*=-0.12  *p*=0.547  *p_adjusted_*=0. 995 | *r*=-0.06  *p*=0.751  *p_adjusted_*=0. 995 | *r*=--0.02  *p*=0.944  *p_adjusted_*=0.995 | *r*=0.02  *p*=0.914  *p_adjusted_*=0.831 |  |
|  | mIns/tCR in gray matter | *r*=0.42  *p*=0.024  *p_adjusted_*=0.360 | *r*=0.13  *p*=0.509  *p_adjusted_*=0. 995 | *r*=0.02  *p*=0.938  *p_adjusted_*=0. 995 | *r*=0.27  *p*=0.179  *p_adjusted_*=0. 831 | *r*=0.05  *p*=0.833  *p_adjusted_*=0. 995 | *r*=0.02  *p*=0.929  *p_adjusted_*=0. 995 |  |
|  | mIns/tCR in white matter | *r*=0.26  *p*=0.179  *p_adjusted_*=0.831 | *r*=0.01  *p*=0.949  *p_adjusted_*=0. 995 | *r*=0.00  *p*=0.995  *p_adjusted_*=0. 995 | *r*=0.02  *p*=0.912  *p_adjusted_*=0. 995 | *r*=0.07  *p*=0.752  *p_adjusted_*=0. 995 | *r*=-0.09  *p*=0.666  *p_adjusted_*=0. 995 |  |
| HC | tNAA/tCR in gray matter | *r*=0.14  *p*=0.432  *p_adjusted_*=0.663 | *r*=-0.29  *p*=0.129  *p_adjusted_*=0.663 | *r*=0.07  *p*=0.727  *p_adjusted_*=0.826 | *r*=0.20  *p*=0.31  *p_adjusted_*=0.663 | *-* | *r*=-0.04  *p*=0.825  *p_adjusted_*=0.873 |  |
|  | tCho/tCR in gray matter | *r*=-0.13  *p*=0.511  *p_adjusted_*=0.663 | *r*=0.23  *p*=0.232  *p_adjusted_*=0.663 | *r*=-0.17  *p*=0.389  *p_adjusted_*=0.663 | *r*=-0.14  *p*=0.468  *p_adjusted_*=0.663 | *-* | *r*=-0.14  *p*=0.493  *p_adjusted_*=0.663 |  |
|  | Glx/tCR in gray matter | *r*=-0.04  *p*=0.838  *p_adjusted_*=0.873 | *r*=0.16  *p*=0.412  *p_adjusted_*=0.663 | *r*=-0.01  *p*=0.942  *p_adjusted_*=0.942 | *r*=-0.39  *p*=0.038  *p_adjusted_*=0.475 | *-* | *r*=0.29  *p*=0.136  *p_adjusted_*=0.993 |  |
|  | mIns/tCR in gray matter | *r*=0.19  *p*=0.333  *p_adjusted_*=0.663 | *r*=-0.09  *p*=0.700  *p_adjusted_*=0.826 | *r*=-0.12  *p*=0.530  *p_adjusted_*=0.663 | *r*=-0.22  *p*=0.266  *p_adjusted_*=0.663 | *-* | *r*=0.13  *p*=0.510  *p_adjusted_*=0.663 |  |
|  | mIns/tCR in white matter | *r*=0.40  *p*=0.03  *p_adjusted_*=0.475 | *r*=-0.25  *p*=0.192  *p_adjusted_*=0.663 | *r*=-0.19  *p*=0.324  *p_adjusted_*=0.663 | *r*=-0.20  *p*=0.318  *p_adjusted_*=0.663 | *-* | *r*=0.27  *p*=0.179  *p_adjusted_*=0.663 |  |

Pearson correlation coefficients *r* are reported for each group. Plasma leptin concentrations were log-transformed prior to analysis due to deviations from normality. Plasma leptin concentrations below the limit of detection of 0.05 μg/l were imputed as (limit of detection)/√2. Reported are raw *p* values and *p* values adjusted for multiple comparisons (30 tests in the AN group, 25 tests in the HC group) using the False Discovery Rate (FDR) correction method of Benjamini and Hochberg ^4^. Asterisks denote a significant group difference after multiple comparison correction: *=*p*<0.05, **=*p*<0.01, ***=*p*<0.001. Abbreviations: AN, patients with anorexia nervosa; BMI-SDS, standardized body mass index; Glx, glutamine and glutamate concentrations (pooled signal); HC, healthy control participants; tCho, total choline concentration (contributions of glycerophosphocholine, phosphocholine and free choline, pooled signal); tNAA, total N-acetyl-aspartate concentration (contributions of N-acetyl aspartate and N-acetyl-aspartyl-glutamate, pooled signal).

**Table S2. Replication analyses of group comparisons of total N-acetyl-aspartate, total choline, glutamat/glutamin and myoinositol concentrations relative to total creatin (tCr) without participants with binge-purge subtype (N=1) or psychiatric comorbidities (N=6)**

|  |  | Group, mean (SD) | | Analyses | | | |
| --- | --- | --- | --- | --- | --- | --- | --- |
|  |  | AN | HC | *t* | *df* | *p/p_adjusted_* | *Cohen’s d* |
| Exclusion participant with binge-purge subtype (N=1) | tNAA/tCr in gray matter | 1.21 (0.20) | 1.38 (0.16) | -3.58 | 53.61 | <0.001/<0.001*** | 0.95 |
|  | tCho/tCr in gray matter | 0.33 (0.04) | 0.27 (0.04) | 5.85 | 55.44 | <0.001/<0.001*** | -1.55 |
|  | Glx/tCr in gray matter | 2.18 (0.19) | 1.98 (0.23) | 3.62 | 56.19 | <0.001/0.001*** | -0.96 |
|  | mIns/tCr in gray matter | 0.86 (0.08) | 0.91 (0.07) | -2.89 | 54.68 | 0.005/0.005** | 0.77 |
|  | mIns/tCr in white matter | 0.73 (0.08) | 0.81 (0.10) | -3.10 | 54.36 | 0.003/0.004** | 0.82 |
| Exclusion participants with psychiatric comorbidities (N=6) | tNAA/tCr in gray matter | 1.22 (0.20) | 1.38 (0.16) | -3.04 | 43.02 | 0.002/<0.003** | 0.86 |
|  | tCho/tCr in gray matter | 0.34 (0.04) | 0.27 (0.04) | 5.95 | 45.45 | <0.001/<0.001*** | -1.67 |
|  | Glx/tCr in gray matter | 2.20 (0.20) | 1.98 (0.23) | 3.70 | 51.70 | <0.001/0.001*** | -1.02 |
|  | mIns/tCr in gray matter | 0.87 (0.07) | 0.91 (0.07) | -2.39 | 48.08 | 0.021/0.021* | 0.67 |
|  | mIns/tCr in white matter | 0.74 (0.08) | 0.81 (0.1) | -2.64 | 51.98 | 0.011/0.014* | 0.73 |

Asterisks denote a significant group difference: *=*p*<0.05, **=*p*<0.01, ***=*p*<0.001. Abbreviations: AN, patients with anorexia nervosa; HC, healthy control participants; tCho, total choline concentration (contributions of glycerophosphocholine, phosphocholine and free choline, pooled signal); tNAA, total N-acetyl-aspartate concentration (contributions of N-acetyl aspartate and N-acetyl-aspartyl-glutamate, pooled signal),Glx, gluatamat and glutamin, mIns, myoinositol, tCr, total creatin (contributions of creatine and phosphocreatine) used to calculate ratios.

**References cited here:**

1 Lin A, Andronesi O, Bogner W, Choi I-Y, Coello E, Cudalbu C *et al.* Minimum reporting standards for in vivo magnetic resonance spectroscopy (MRSinMRS): experts’ consensus recommendations. *NMR Biomed* 2021; **34**: e4484.

2 Destrieux C, Fischl B, Dale A, Halgren E. Automatic parcellation of human cortical gyri and sulci using standard anatomical nomenclature. *Neuroimage* 2010; **53**: 1–15.

3 Bernardoni F, King JA, Geisler D, Stein E, Jaite C, Nätsch D *et al.* Weight restoration therapy rapidly reverses cortical thinning in anorexia nervosa: A longitudinal study. *Neuroimage* 2016; **130**: 214–222.

4 Benjamini Y, Hochberg Y. Controlling the false discovery rate: a practical and powerful approach to multiple testing. *J R Stat Soc B Met* 1995; **57**: 289–300.
